# Artificial Intelligence-Enabled Comprehensive Electronic Health Record Phenotyping at a Large Scale

**DOI:** 10.1093/geroni/igaf122.2241

**Published:** 2025-12-31

**Authors:** Niels Turley, Marta Fernandes, Aditya Gupta, Manohar Ghanta, Haoqi Sun, Robert Thomas, Sahar Zafar, M Brandon Westover

**Affiliations:** Beth Israel Deaconess Medical Center, Boston, Massachusetts, United States; Massachusetts General Hospital, Boston, Massachusetts, United States; Beth Israel Deaconess Medical Center, Boston, Massachusetts, United States; Beth Israel Deaconess Medical Center, Boston, Massachusetts, United States; Beth Israel Deaconess Medical Center, Boston, Massachusetts, United States; Beth Israel Deaconess Medical Center, Boston, Massachusetts, United States; Massachusetts General Hospital, Boston, Massachusetts, United States; Beth Israel Deaconess Medical Center, Boston, Massachusetts, United States

## Abstract

The Electronic health record (EHR) contains rich and ever-growing information, especially for the gerontologic population with multiple comorbidities. With the advent of powerful artificial intelligence (AI) tools, we can perform accurate EHR phenotyping, which is the foundation for downstream analyses. Here, we performed EHR phenotyping of ten diseases in a large multi-site clinical dataset of 145,787 unique patients, including epilepsy (and subtypes), ischemic stroke, subarachnoid hemorrhage, subdural hematoma, Alzheimer’s diseases and related dementias, Parkinson’s disease, cardiac arrest, traumatic brain injury, brain tumor, and congestive heart failure. We used AI-enabled natural language processing that extracts the presence of keywords from unstructured clinical notes while considering negations, as well as structured diagnosis codes (ICD) and medications. We used logistic regression, random forest, and XGBoost. The dataset was constructed from two sites by sampling an equal number of participants in people with or without ICD or medication in the EHR, which forms four groups: ICD+Med+, ICD+Med-, ICD-Med+, and ICD-Med-. We manually annotated each case as positive or negative by reading the clinical notes. In this way, the dataset has a roughly balanced positive/negative ratio to ease training. We then did leave-one-site-out cross-validation. The areas under the receiver operator curve are higher than 0.95 across the diseases. The areas under the precision-recall curve are higher than 0.83 across the diseases. For each disease, we conducted detailed error analyses for both false positives and negatives. The results lead to accurate phenotyping and insights from patient phenotypes at both population and individual levels.

